# Deaths of despair in England, 2014–2022: trends by sex and socioeconomic deprivation before and after COVID-19

**DOI:** 10.3389/ijph.2026.1609841

**Published:** 2026-09-01

**Authors:** Steven Wyatt, Jonathan Spencer, Paul Seamer, Andrew Jones, Alison Turner, Mai Stafford, Kathryn Marszalek, Mohammed Amin Mohammed

**Affiliations:** 1 The Strategy Unit, Birmingham, United Kingdom; 2 The Health Foundation, London, United Kingdom; 3 Brent Council, Wembley, United Kingdom; 4 Faculty of Health Studies, University of Bradford, Bradford, United Kingdom; 5 Cardiff University Business School, Cardiff, United Kingdom

**Keywords:** COVID-19 pandemic, deaths of despair, health inequalities, mortality trends, socioeconomic deprivation

## Abstract

**Objectives:**

Deaths of despair—alcohol, substance use, suicide and non-organic mental health related—have attracted international attention, but English evidence remains limited. We examined trends in deaths of despair in England (2014–2022), including COVID-19 impacts and inequalities by sex and deprivation.

**Methods:**

We analysed all registered deaths in England (2014–2022), identifying deaths of despair by ICD-10 code. Age-standardised mortality rates, years of life lost, and inequalities by sex and deprivation were calculated. Log-linear regression estimated pre-pandemic trends and deviations during COVID-19.

**Results:**

There were 133,600 deaths of despair (2.9% of all deaths). Age-standardised mortality rates rose 33%, from 22.6 to 30.1 per 100,000. Mortality was consistently higher in men and deprived areas, with widening inequalities. During COVID-19, mortality temporarily exceeded the pre-pandemic trend, particularly among women and deprived groups, before returning to trend. Deaths of despair accounted for a rising share of years of potential life lost (13.9%–16.6%).

**Conclusion:**

Deaths of despair increased substantially in England, contributing a growing burden of premature mortality. Persistent, widening inequalities highlight a public health challenge requiring surveillance and interventions addressing immediate causes and social determinants.

## Introduction

Deaths of despair refer to fatalities caused by suicide, drug overdoses and alcohol-related diseases, often linked to a range of social and economic factors including unemployment, poverty and eroding community ties. The term, popularised by economists Case and Deaton in 2015 [1], highlighted rising mortality in the United States (US), particularly among middle-aged, less-educated White individuals since the late 20th century [2, 3]. The theoretical framework underlying this phenomenon centres on economic dislocation—including the collapse of manufacturing employment, wage stagnation and erosion of working-class identity and social status following deindustrialisation in the US from the 1970s onwards. This structural decline is thought to generate cumulative disadvantage across the life course, manifesting in hopelessness, loss of purpose and community fragmentation. While deaths of despair are commonly attributed to hopelessness, isolation and chronic stress arising from economic dislocation and weakened social safety nets, others argue that the availability and lethality of intoxicants—particularly prescription opioids—play a more central role (the supply-side hypothesis) [4]. The phenomenon therefore highlights the complex interplay between physical and mental health, economic insecurity, cultural trends and broader societal wellbeing.

A growing literature has examined deaths of despair [4–20]. Recent studies suggest that rising deaths of despair are not confined to the original US population described by Case and Deaton but are also evident across other population groups and ethnicities [2, 3]. Several studies have examined whether the patterns observed in the US are also present in other high-income countries [21–24]. Although many countries have experienced increasing deaths of despair, the US remains unusual in both the scale and pace of the increase, reflecting the combined effects of economic dislocation, a severe opioid epidemic fuelled in part by aggressive pharmaceutical marketing, and comparatively weaker social welfare and healthcare systems. By contrast, many Western European countries have experienced more modest increases despite facing similar underlying socioeconomic pressures.

Analysis by the Institute for Fiscal Studies in 2019 found that although deaths of despair occurred at lower rates in the UK than in the US, they were nevertheless increasing [25]. A 2024 ecological study estimated that approximately 15,000 people died from diseases of despair each year in England between 2019 and 2021, representing around 2.9% of all deaths [26]. That study demonstrated a strong association with socioeconomic deprivation but was unable to examine how inequalities changed over time or differed by sex. More recently, attention has focused on the potential impact of the COVID-19 pandemic, which disrupted healthcare services, employment, social connectedness and economic security, all of which may plausibly influence deaths of despair [27, 28]. However, international evidence remains mixed, with important differences between countries and causes of death [29].

In this descriptive study, we extend the existing literature by examining national trends in deaths of despair in England between 2014 and 2022, spanning the pre-pandemic, pandemic and post-pandemic periods. Specifically, we quantify trends in age-standardised mortality rates, years of potential life lost, and inequalities by sex and socioeconomic deprivation, providing a comprehensive description of how these deaths evolved before, during and after the COVID-19 pandemic.

## Methods

### Data sources

We used the Office for National Statistics (ONS) Mortality dataset as our primary data source [30]. The dataset contains person-level pseudonymised information on all registered deaths in England, including the date of death, month and year of birth, sex, Lower Super Output Area (LSOA) of residence, and the underlying cause of death coded using the International Classification of Diseases, Tenth Revision (ICD-10). Our analysis covered deaths registered between 1 January 2014 and 31 December 2022. We excluded a small number of records (0.003%; 126 of 4,649,831) where sex was missing.

Mid-year population estimates by age, sex and LSOA were obtained from the Office for National Statistics [31].

### Variables

Deaths of despair were identified from the underlying cause of death recorded on the death certificate using ICD-10 codes. Deaths of despair comprised four groups: (1) suicide and injuries or poisonings of undetermined intent; (2) substance misuse; (3) alcohol misuse; and (4) non-organic mental health-related deaths. All remaining deaths were classified as non-despair deaths and used as a comparator.

There is no universally accepted definition of deaths of despair, and published studies differ in the conditions included. We adopted the same ICD-10 classification as our companion study of hospital admissions for conditions of despair to ensure direct comparability between mortality and hospitalisation analyses across the two studies. The complete list of ICD-10 codes is provided in Supplementary Table S1 to facilitate comparison with alternative definitions used in the literature.

Socioeconomic deprivation was measured using the English Index of Multiple Deprivation (IMD) 2019 [32]. The IMD is a small-area composite measure based on seven domains: income, employment, education, health, crime, barriers to housing and services, and the living environment. Individuals were assigned an IMD score according to their LSOA of residence. LSOAs are small geographical areas in England containing approximately 1,500 residents that are designed for reporting small-area statistics. LSOAs were ranked nationally according to IMD score and grouped into population-weighted deprivation quintiles, ranging from the most deprived (quintile 1) to the least deprived (quintile 5).

### Statistical methods

Age at death was calculated using the date of death together with the month and year of birth. Age was imputed for a small number of records (0.03%; 1,459 of 4,649,705) where month and year of birth were incomplete using a k-nearest neighbours algorithm (k = 1) based on the underlying cause of death group [33, 34]. An additional random variable was included in the matching process to minimise repeated selection of the same donor record.

Annual mortality rates by deprivation quintile were directly age-sex standardised, while mortality rates by sex were directly age standardised. The England population in 2014 was used as the standard population because it represented the first year of the study period. Standardisation was undertaken using single-year age groups (0–89 years and ≥90 years). Confidence intervals for directly standardised rates were calculated using the Chiang-Keyfitz method [35, 36].

Annual absolute and relative differences in directly standardised mortality rates were calculated between men and women and between the most and least deprived quintiles. Relative inequalities were defined as the ratio of directly standardised mortality rates between the groups being compared. Confidence intervals for these differences were estimated using non-parametric bootstrapping with 10,000 replications because we were unable to identify a closed-form variance estimator for the absolute and relative differences between directly standardised mortality rates.

**FIGURE 1:**
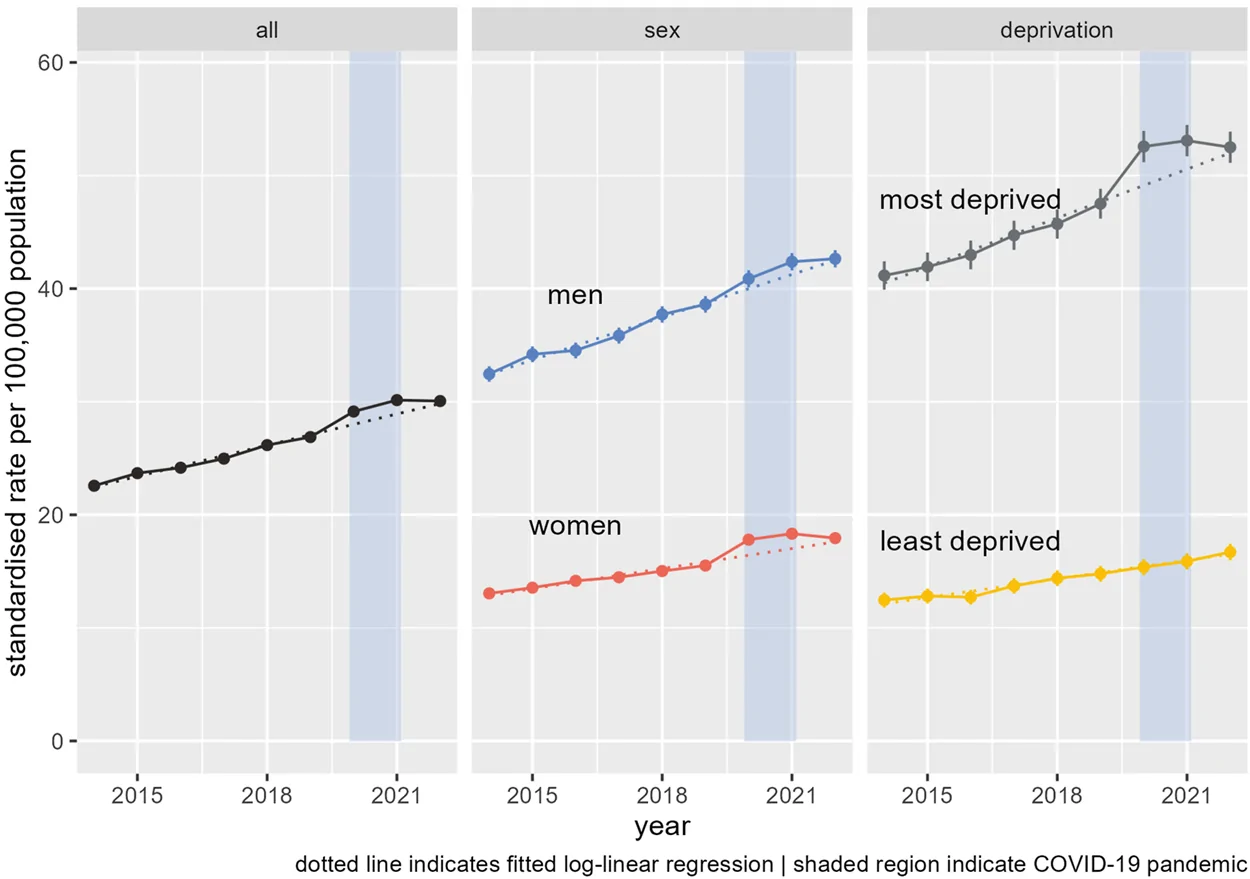
Directly age-standardised mortality rates for deaths of despair per 100,000 population overall, by sex and socioeconomic deprivation (England, 2014–2022).

To estimate the impact of the COVID-19 pandemic on mortality rates and inequalities, we fitted log-linear regression models with calendar year as the only explanatory variable. The years 2020 and 2021 were excluded from estimation of the secular trend because they represented the period of greatest disruption associated with the COVID-19 pandemic in England. The fitted models therefore estimated the underlying pre-pandemic trend. Model coefficients are provided in Supplementary Table S2. The fitted regression lines shown in Figure 1 represent these estimated pre-pandemic secular trends, against which observed mortality during the COVID-19 pandemic was compared. Log-linear regression models estimate proportional (percentage) changes over time, whereas linear regression models estimate absolute changes. We selected log-linear regression because mortality rates were expected to change proportionally over time, which was consistent with the descriptive aims of the study.

Four sensitivity analyses were undertaken. First, we repeated the primary analysis (Figure 1) including deaths due to injuries and poisonings of undetermined intent (Supplementary Figure S1). Secondly, we undertook stratified analyses examining the interaction between sex and socioeconomic deprivation (Supplementary Figure S2). Thirdly, we repeated the primary analysis after excluding deaths among people aged 90 years or over to assess whether increasing longevity influenced the observed trends. Finally, we repeated the analyses after excluding deaths attributed to non-organic mental health conditions, reflecting an alternative definition of deaths of despair used in some previous studies. The latter two sensitivity analyses are presented in Supplementary Figure S3.

Years of potential life lost (YPLL) were calculated by summing, for individuals dying before 75 years of age, the difference between age at death and 75 years [37].

Data preprocessing included filtering, summarising and joining the raw datasets, deriving age at death, assigning deprivation quintiles, constructing analytical datasets and undertaking quality assurance checks prior to statistical analysis. Data preprocessing was undertaken using SQL Server Management Studio version 18.7.1. Statistical analyses were conducted in R version 4.3.1 [38]. The analytical code developed for this study is publicly available through a GitHub repository [39].

### Literature search strategy

Our search strategy for identifying relevant published literature is described in Supplementary Table S3.

## Results

### Characteristics of deaths

Between 2014 and 2022, there were approximately 133,600 deaths of despair in England, accounting for 2.9% of all registered deaths (Table 1). Deaths of despair were more common among men, younger adults and people living in more socioeconomically deprived areas. Nearly seven in ten deaths of despair (69.8%) occurred in men, while among adults aged 18–34 years, deaths of despair accounted for 38.7% of all deaths.

**TABLE 1:** Characteristics of deaths by year, sex, age group, and deprivation quintile | England 2014–2022.

Characteristic	Deaths of despair		Other deaths		​
​	n (000s)	%	n (000s)	%	Deaths of despair as a share of all deaths
Total	133.6	100.0%	4,516.1	100.0%	2.9%
Sex					
Female	40.4	30.2%	2,295.1	50.8%	1.7%
Male	93.2	69.8%	2,220.9	49.2%	4.0%
Age group					
<18 years	1.0	0.7%	32.4	0.7%	2.9%
18–34 years	20.2	15.1%	32.0	0.7%	38.7%
35–54 years	60.1	45.0%	203.0	4.5%	22.9%
55–74 years	43.3	32.4%	1,090.0	24.1%	3.8%
75+ years	9.0	6.8%	3,158.6	69.9%	0.3%
Deprivation quintile					
Quintile 1 - most deprived	44.0	32.9%	924.0	20.5%	4.5%
Quintile 2	30.5	22.8%	900.4	19.9%	3.3%
Quintile 3	23.9	17.9%	927.6	20.5%	2.5%
Quintile 4	19.3	14.4%	912.0	20.2%	2.1%
Quintile 5 – least deprived	15.3	11.4%	843.0	18.7%	1.8%
Not known	0.6	0.5%	9.0	0.2%	6.5%
Year					
2014	12.3	9.2%	458.4	10.2%	2.6%
2015	13.0	9.8%	481.5	10.7%	2.6%
2016	13.4	10.0%	483.6	10.7%	2.7%
2017	13.9	10.4%	489.7	10.8%	2.8%
2018	14.7	11.0%	490.4	10.9%	2.9%
2019	15.2	11.4%	486.0	10.8%	3.0%
2020	16.5	12.4%	555.1	12.3%	2.9%
2021	17.2	12.9%	535.9	11.9%	3.1%
2022	17.3	13.0%	535.6	11.9%	3.1%

The burden of deaths of despair was strongly associated with socioeconomic deprivation. Almost one-third (32.9%) of all deaths of despair occurred among people living in the most deprived quintile of areas, compared with 11.4% in the least deprived quintile. Deaths of despair accounted for 4.5% of all deaths in the most deprived areas compared with 1.8% in the least deprived areas.

The number of deaths of despair increased by 40.7% over the study period, from 12,300 deaths in 2014 to 17,300 deaths in 2022, substantially exceeding the 16.8% increase observed for all other causes of death (Table 1).

Across the study period, alcohol-related deaths accounted for the largest number of deaths of despair, followed by suicide, substance misuse and non-organic mental health-related deaths (Supplementary Table S4). Between 2014 and 2022, deaths attributable to alcohol use increased by 56.2%, while deaths related to substance misuse increased by 56.9%. By comparison, suicide deaths increased more modestly (14.0%), whereas non-organic mental health-related deaths remained comparatively uncommon throughout the study period.

Deaths due to injuries or poisonings of undetermined intent comprised 5.2% of all deaths of despair (Supplementary Table S5). Their contribution declined from 7.6% of deaths of despair in 2014 to 3.0% in 2022, with similar reductions observed among both men and women and across deprivation quintiles. This indicates that the observed increase in deaths of despair was unlikely to be explained by changes in the contribution of deaths classified as undetermined intent.

### Mortality rate trends

Directly standardised mortality rates for deaths of despair increased from 22.6 per 100,000 population (95% CI 22.2–23.0) in 2014 to 30.1 per 100,000 (95% CI 29.6–30.5) in 2022, equivalent to an average annual increase of 3.6% (Figure 1). Throughout the study period, mortality rates remained consistently higher among men than women and among people living in the most deprived compared with the least deprived areas (Table 2). (Annual standardised rates for all deprivation quintiles are available in Supplementary Table S6 in the Supplementary Material).

**TABLE 2:** Directly standardised rates of deaths of despair in total, by sex and deprivation quintile (95% confidence interval) | England 2014–2022.

​	​	Sex**		Deprivation quintile*	
year	All*	Female	Male	Most deprived	Least deprived
2014	22.6 (22.2, 23.0)	13.1 (12.6, 13.5)	32.5 (31.8, 33.1)	41.2 (39.9, 42.4)	12.5 (11.8, 13.1)
2015	23.7 (23.3, 24.1)	13.6 (13.1, 14.0)	34.2 (33.5, 34.9)	41.9 (40.7, 43.2)	12.8 (12.2, 13.5)
2016	24.2 (23.8, 24.6)	14.2 (13.7, 14.6)	34.5 (33.8, 35.2)	43.0 (41.7, 44.2)	12.7 (12.1, 13.4)
2017	25.0 (24.5, 25.4)	14.5 (14.0, 14.9)	35.8 (35.1, 36.6)	44.7 (43.4, 46.0)	13.7 (13.0, 14.4)
2018	26.2 (25.7, 26.6)	15.0 (14.6, 15.5)	37.7 (37.0, 38.4)	45.7 (44.4, 47.0)	14.4 (13.7, 15.1)
2019	26.9 (26.4, 27.3)	15.5 (15.1, 16.0)	38.6 (37.9, 39.3)	47.5 (46.2, 48.8)	14.8 (14.1, 15.5)
2020	29.1 (28.7, 29.6)	17.8 (17.3, 18.3)	40.9 (40.1, 41.6)	52.6 (51.2, 54.0)	15.4 (14.7, 16.1)
2021	30.1 (29.7, 30.6)	18.3 (17.8, 18.8)	42.4 (41.6, 43.1)	53.1 (51.7, 54.5)	15.9 (15.2, 16.6)
2022	30.1 (29.6, 30.5)	17.9 (17.5, 18.4)	42.6 (41.9, 43.4)	52.5 (51.1, 53.9)	16.7 (16.0, 17.4)

Per 100,000 population, *age-sex standardised, **age standardised.

Observed mortality rates exceeded the underlying pre-pandemic secular trend during 2020 and 2021, before returning towards the expected trajectory in 2022. Overall mortality rates were 4.1% above the expected trend in 2020 (95% CI 2.5%–5.7%) and 4.2% above trend in 2021 (95% CI 2.7%–5.8%). These departures from the expected trend were more pronounced among women than men and among people living in the most deprived areas. In contrast, mortality rates in the least deprived areas did not differ significantly from the expected trend during the pandemic years (Figure 1).

Supplementary Figure S1 shows the equivalent analysis after including deaths due to injuries or poisonings of undetermined intent. The similarity of the findings indicates that inclusion of these deaths does not materially affect the overall conclusions.

**FIGURE 2:**
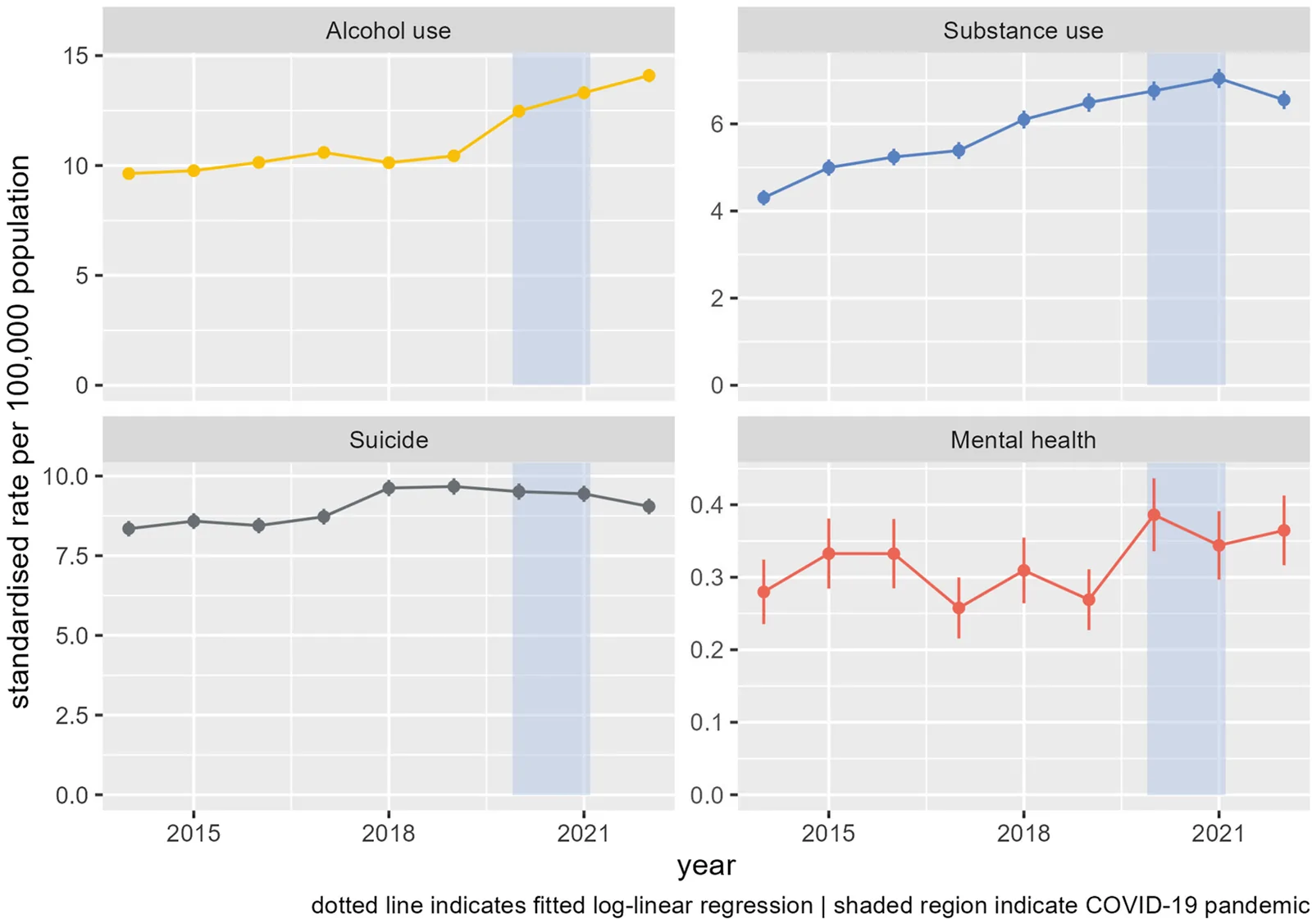
Directly age-standardised mortality rates for the four components of deaths of despair per 100,000 population (England, 2014–2022).

Patterns differed across the four components of deaths of despair (Figure 2; Supplementary Table S7). Alcohol-related mortality increased gradually before rising sharply during the COVID-19 pandemic and continued to increase thereafter. Mortality associated with substance misuse increased steadily throughout the study period, with little evidence of a distinct pandemic effect. Suicide mortality increased until 2018 before declining modestly, while mortality attributed to non-organic mental health conditions remained low throughout the study period.

### Inequalities by sex and socioeconomic deprivation

**FIGURE 3:**
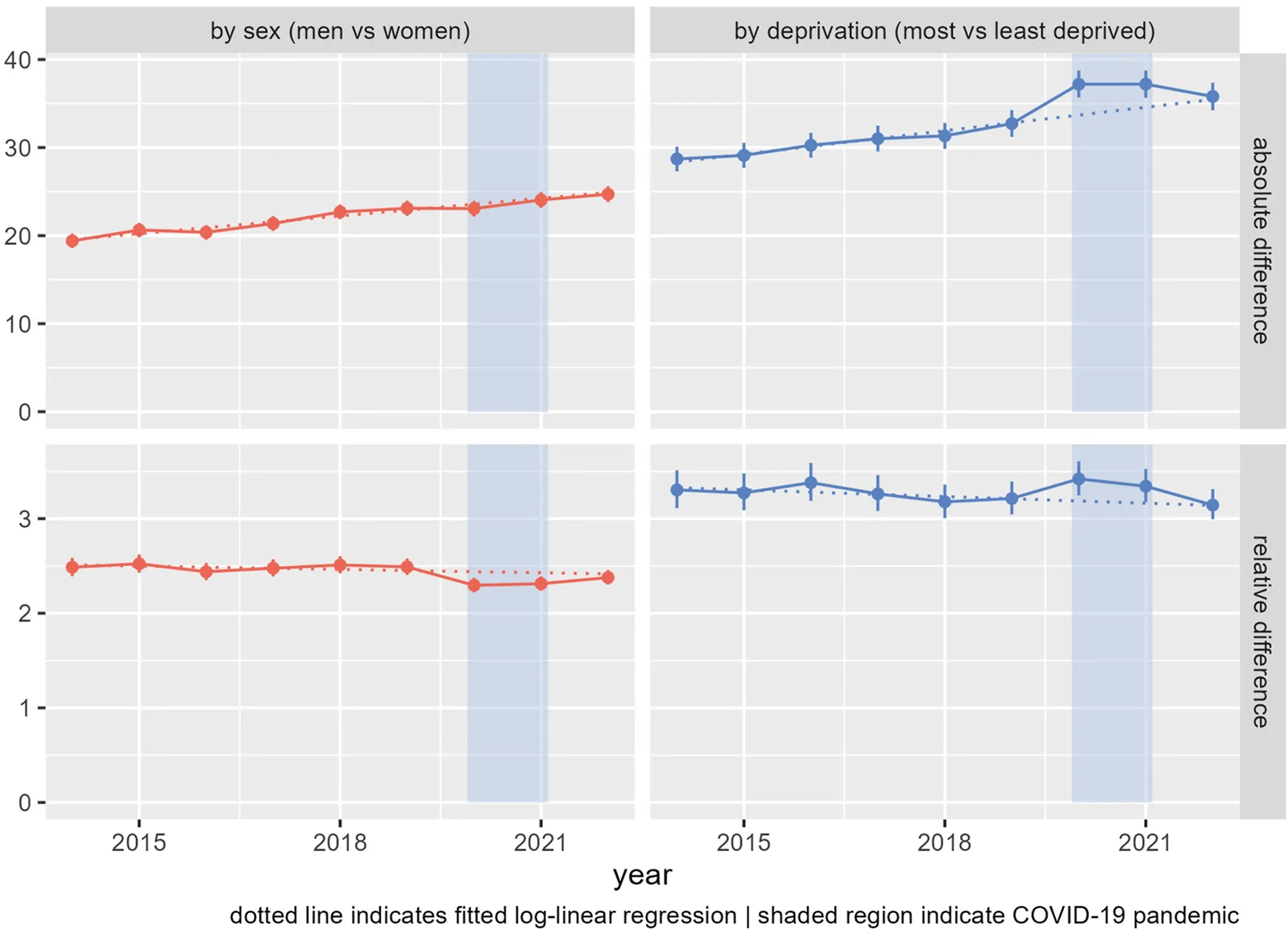
Absolute and relative inequalities in directly age-standardised mortality rates for deaths of despair by sex and socioeconomic deprivation (England, 2014–2022).

Absolute inequalities increased over the study period for both sex and socioeconomic deprivation (Figure 3). The absolute difference in mortality rates between men and women increased from 19.4 per 100,000 population (95% CI 18.6–20.2) in 2014 to 24.7 per 100,000 (95% CI 23.8–25.6) in 2022. The corresponding absolute difference between the most and least deprived quintiles increased from 28.7 per 100,000 (95% CI 27.3–30.1) to 35.8 per 100,000 (95% CI 34.2–37.4). Relative inequalities by both sex and deprivation changed comparatively little over the study period.

The COVID-19 pandemic was associated with a temporary widening of socioeconomic inequalities. Both the absolute and relative differences between the most and least deprived areas increased significantly above the underlying secular trend during 2020 and 2021. By contrast, relative differences between men and women narrowed during the pandemic, while absolute differences by sex showed no significant departure from the pre-pandemic trend (Figure 3).

Supplementary Figure S2 extends these analyses by examining the interaction between sex and socioeconomic deprivation. The deprivation gradient in deaths of despair was consistently steeper among men than women throughout the study period, although this difference narrowed modestly over time.

### Years of potential life lost

Deaths of despair contributed disproportionately to premature mortality because they occurred at younger ages than most other causes of death. The total number of years of potential life lost increased from approximately 310,000 years in 2014 to 409,000 years in 2022 (Table 3). Consequently, deaths of despair accounted for an increasing share of all years of potential life lost, rising from 13.9% in 2014 to 16.6% in 2022. This increase primarily reflects the rising number of deaths of despair over the study period rather than a substantial change in the age at which these deaths occurred.

**TABLE 3:** Years of potential life lost (thousands) to deaths of despair in total, by sex and deprivation quintile | England 2014–2022.

​	​	Sex		Deprivation	
year	All	Female	Male	Most deprived	Least deprived
2014	310 (13.9%)	87 (9.7%)	223 (16.7%)	113 (16.7%)	94 (5.7%)
2015	329 (14.4%)	90 (10.0%)	239 (17.2%)	115 (16.7%)	100 (6.0%)
2016	339 (14.4%)	96 (10.1%)	243 (17.3%)	120 (16.7%)	99 (5.7%)
2017	352 (15.1%)	99 (10.8%)	253 (17.8%)	124 (17.5%)	106 (6.1%)
2018	374 (15.8%)	104 (11.1%)	270 (18.9%)	131 (18.3%)	111 (6.3%)
2019	383 (16.5%)	107 (11.7%)	275 (19.5%)	134 (19.0%)	116 (6.8%)
2020	409 (16.2%)	123 (12.5%)	286 (18.6%)	149 (18.8%)	121 (6.5%)
2021	418 (16.0%)	125 (12.3%)	293 (18.3%)	148 (18.0%)	127 (6.7%)
2022	409 (16.6%)	121 (12.5%)	288 (19.2%)	142 (18.6%)	134 (7.5%)

%s indicate the share of years of life lost due to deaths of despair.

## Discussion

### Main findings

This descriptive study examined temporal trends and inequalities in deaths of despair in England between 2014 and 2022, with particular emphasis on differences by sex and socioeconomic deprivation, and on the impact of the COVID-19 pandemic. Deaths of despair increased substantially over the study period, with persistent and widening inequalities. Men and people living in the most deprived areas consistently experienced the highest mortality rates. During the pandemic, however, the gap between men and women narrowed as mortality among women increased more rapidly, while inequalities between the most and least deprived areas widened. By 2022, mortality rates had returned towards the underlying pre-pandemic trend, although at a higher overall level than before the pandemic. Deaths of despair also accounted for an increasing proportion of years of potential life lost, highlighting their disproportionate contribution to premature mortality.

### Comparison with existing literature

Our findings are consistent with a growing international literature showing that deaths of despair have increased over the past decade, although the magnitude and underlying drivers vary between countries. The original work of Case and Deaton described substantial increases in deaths of despair among middle-aged White Americans, while subsequent studies have shown that similar patterns are evident across the UK and parts of Europe, albeit with important differences in scale and causes [21–24, 26, 40].

The present study extends previous UK evidence in several important ways. First, it provides contemporary national estimates covering the period to 2022, including the COVID-19 pandemic. Secondly, it demonstrates that inequalities by socioeconomic deprivation have widened over time, with the pandemic disproportionately affecting people living in the most deprived areas. Thirdly, by examining years of potential life lost, our findings highlight that deaths of despair contribute disproportionately to premature mortality despite representing a relatively small proportion of all deaths.

Our findings are also consistent with previous studies reporting higher mortality rates among men and increasing inequalities associated with socioeconomic deprivation [24, 26, 41]. While international studies have reported increases in deaths of despair during the COVID-19 pandemic [27–29], our analysis adds important detail by demonstrating that the pandemic was associated with a temporary widening of socioeconomic inequalities, whereas differences between men and women narrowed because mortality increased more rapidly among women during this period.

### Interpretation of the findings

The observed increase in deaths of despair is likely to reflect the combined influence of long-term structural factors and more acute societal shocks. Previous research has linked deaths of despair to economic insecurity, unemployment, austerity, social dislocation and declining community cohesion, although the relative importance of these mechanisms remains debated [19–21, 25, 26]. Our findings are consistent with this broader literature, showing that mortality increased steadily over the study period while remaining concentrated among people living in the most deprived areas.

The COVID-19 pandemic appears to have exacerbated these underlying trends rather than fundamentally altering them. Overall mortality temporarily exceeded the expected pre-pandemic trajectory, with the largest increases occurring among women and among people living in the most deprived areas. This pattern is consistent with evidence that the pandemic disproportionately affected socially and economically disadvantaged populations through healthcare disruption, financial insecurity, bereavement, and changes in the availability and use of alcohol and drugs [27–29].

Although our study was not designed to identify causal mechanisms, the persistence and widening of socioeconomic inequalities suggests that deaths of despair should be understood within a broader social and economic context rather than as isolated manifestations of individual mental illness or substance misuse.

### Implications

Our findings demonstrate that deaths of despair are an increasingly important contributor to premature mortality in England and that the burden falls disproportionately on people living in more deprived areas. The persistence of these inequalities before, during and after the COVID-19 pandemic suggests that deaths of despair reflect longstanding structural and socioeconomic disadvantage rather than a temporary consequence of the pandemic alone. These findings reinforce the need for coordinated public health approaches that address the wider social determinants of health alongside evidence-based interventions targeting alcohol misuse, substance use and suicide prevention [42, 43]. Further research is needed to better understand the mechanisms underlying these trends and to evaluate interventions designed to reduce deaths of despair.

### Strengths and limitations

This study has several strengths. We analysed all registered deaths in England over a nine-year period using nationally complete mortality data and employed directly age-standardised mortality rates to allow meaningful comparisons over time and between population groups. We also examined years of potential life lost, providing additional insight into the burden of premature mortality associated with deaths of despair.

Finally, while the study identifies important temporal patterns and inequalities, these findings should be interpreted within the context of the descriptive study design.

### Conclusion

Deaths of despair increased substantially in England between 2014 and 2022 and now account for a disproportionate share of premature mortality. Persistent and widening socioeconomic inequalities indicate that the burden falls most heavily on people living in deprived communities, while the COVID-19 pandemic temporarily amplified these inequalities. These findings highlight deaths of despair as an important and growing public health challenge and underscore the need for continued surveillance together with rigorous evaluation of interventions that address both the immediate causes of these deaths and their wider social and economic determinants.
