# Supplementary material for: Deaths of despair in England, 2014–2022: trends by sex and socioeconomic deprivation before and after COVID-19
Source: Int J Public Health. 2026 Sep 1;71:1609841. doi: 10.3389/ijph.2026.1609841 (PMC13575742; doi:10.3389/ijph.2026.1609841)
Supplement: Supplementary file 1 [file Supplementaryfile1.docx]

# Supplementary Material

**Deaths of despair in England, 2014–2022: trends, inequalities and the impact of the COVID-19 pandemic**

This supplementary material provides additional methodological detail, sensitivity analyses and supporting results that complement the findings presented in the main manuscript.

## Contents

**Supplementary Tables**

- **Supplementary Table S1. ICD-10 code lists used to define deaths of despair.**
- **Supplementary Table S2. Log-linear regression model coefficients.**
- **Supplementary Table S3. Literature search strategy.**
- **Supplementary Table S4. Deaths from injuries or poisonings of undetermined intent as a percentage of all deaths of despair.**
- **Supplementary Table S5. Directly age-standardised mortality rates for deaths of despair by year and socioeconomic deprivation with 95% confidence intervals, England, 2014–2022.**
- **Supplementary Table S6. Directly age-standardised mortality rates for deaths of despair by year and condition group with 95% confidence intervals, England, 2014–2022.**

**Supplementary Figures**

- **Supplementary Figure S1. Directly age-standardised mortality rates for deaths of despair per 100,000 population in England, overall, by sex and socioeconomic deprivation, 2014–2022, using the primary definition and an expanded definition including deaths due to injuries and poisonings of undetermined intent.**
- **Supplementary Figure S2. Directly age-standardised mortality rates for deaths of despair per 100,000 population among men and women, stratified by socioeconomic deprivation, England, 2014–2022.**
- **Supplementary Figure S3. Sensitivity analyses demonstrating the robustness of directly age-standardised mortality rates for deaths of despair per 100,000 population in England, 2014–2022.**

##### Table S1: ICD10 codes used to identify deaths of despair

| Condition group | ICD10 codes | Description |
| --- | --- | --- |
| Substance misuse | F11  F12  F13  F14  F15  F16  F18  F19  X40-X44 | Opioid related disorders  Cannabis related disorders  Sedative, hypnotic, or anxiolytic disorders  Cocaine related disorders  Other stimulant related disorders  Hallucinogen related disorders  Inhalant related disorders  Other psychoactive substance related disorders  Accidental poisonings (drugs and medications) |
| Alcohol misuse | E244  F10  G31.2  G62.1  I42.6  K29.2  K70  K72.1  K73  K74.0  K74.2  K85.2  K86.0  X45 | Alcohol-induced pseudo-Cushing syndrome  Alcohol-related disorders  Degeneration of nervous system due to alcohol  Alcoholic polyneuropathy  Alcoholic cardiomyopathy  Alcoholic gastritis  Alcoholic liver disease  Chronic hepatic failure  Chronic hepatitis, not elsewhere classified  Hepatic fibrosis  Hepatic fibrosis with hepatic sclerosis  Alcohol-induced acute pancreatitis  Alcohol-induced chronic pancreatitis  Accidental poisoning by and exposure to alcohol |
| Suicide, suicide attempt and suicide ideation | X60-X84  Y10-Y34  Y87.0  Y87.2 | Intentional self-harm  Event of undetermined intent  Sequelae of intentional self-harm  Sequelae of event of undetermined intent |
| Mental illness | F20-F29  F30  F31  F32  F33  F34  F39  F39  F40-F48  F50-F59  F60 | Schizophrenia, schizotypal and delusional disorders  Manic episode  Bipolar disorder  Depressive episode  Recurrent depressive episode  Persistent mood [affective] disorders  Other mood disorders  Unspecified mood [affective] disorders  Neurotic, stress-related and somatoform disorders  Behavioural syndromes associated with psychological disturbances and physical factors  Specific personality disorders |

##### Table S2: Log-linear regression model coefficients

| term | estimate | standard error | p value |
| --- | --- | --- | --- |
| mortality rate - all | | | |
| (Intercept) | -14,111 | 553 | 0.000 |
| log(year) | 1,858 | 73 | 0.000 |
| mortality rate - female | | | |
| (Intercept) | -9,061 | 609 | 0.000 |
| log(year) | 1,193 | 80 | 0.000 |
| mortality rate - male | | | |
| (Intercept) | -19,316 | 840 | 0.000 |
| log(year) | 2,543 | 110 | 0.000 |
| mortality rate - most deprived quintile | | | |
| (Intercept) | -22,055 | 1,132 | 0.000 |
| log(year) | 2,904 | 149 | 0.000 |
| mortality rate - least deprived quintile | | | |
| (Intercept) | -8,428 | 692 | 0.000 |
| log(year) | 1,109 | 91 | 0.000 |
| absolute difference in rate (men vs women) | | | |
| (Intercept) | -10,255 | 927 | 0.000 |
| log(year) | 1,351 | 122 | 0.000 |
| relative difference in rate (men vs women) | | | |
| (Intercept) | 183 | 97 | 0.118 |
| log(year) | -24 | 13 | 0.122 |
| absolute difference in rate (most vs least deprived) | | | |
| (Intercept) | -13,627 | 815 | 0.000 |
| log(year) | 1,795 | 107 | 0.000 |
| relative difference in rate (most vs least deprived) | | | |
| (Intercept) | 362 | 126 | 0.035 |
| log(year) | -47 | 17 | 0.036 |

##### Table S3: Search strategy for existing literature

| Sources searched | MEDLINE | 24/09/2024 |
| --- | --- | --- |
|  | Embase | 24/09/2024 |
|  | Goggle Scholar [citation search for Case and Deaton (2015)] | 24/09/2024 |
| Research question | Population | middle aged adults |
|  | Exposure | economic hardship, austerity, unemployment, adversity, economic insecurity (for the purpose of this literature review, papers which cover loneliness or social isolation only have been excluded). |
|  | Outcome of interest | death, hospital admission |
| Inclusion/exclusion criteria | Study types (in scope) | Analysis of mortality or hospitalisations from drug and alcohol poisonings, suicides, and chronic liver diseases driven by “despair” |
|  | Study types (out of scope) | Single centre studies. Commentaries, editorials and letters except for those which discuss methods of analysis. |
|  | Geographical limits (in scope) | Studies from the UK. International comparative studies. Studies from other OECD countries (which report at national level). |
|  | Geographical limits (out of scope) | Studies from other countries which report at local level. |
|  | Date limits | 2015 onwards (since publication of Case and Deaton) |
| MEDLINE search strategy | 1 | ((death* or admission* or hospitalisation* or hospitalization*) adj3 despair).mp. |
|  | 2 | exp Mortality/ |
|  | 3 | exp Hospitalization/ |
|  | 4 | mo.fs. |
|  | 5 | 2 or 3 or 4 |
|  | 6 | exp Suicide/ |
|  | 7 | exp Stress, Psychological/ |
|  | 8 | self-injurious behavior/ or self-mutilation/ |
|  | 9 | exp Liver diseases/ |
|  | 10 | exp Substance-Related Disorders/ |
|  | 11 | exp Mental Disorders/ |
|  | 12 | or/6-11 |
|  | 13 | 5 and 12 |
|  | 14 | Middle Aged/ |
|  | 15 | 13 and 14 |
|  | 16 | (td or ep or sn).fs. |
|  | 17 | 15 and 16 |
|  | 18 | limit 17 to (humans and yr=“2015-Current”) |
|  | 19 | exp sociological factors/ |
|  | 20 | exp socioeconomic factors/ |
|  | 21 | 19 or 20 |
|  | 22 | 17 and 21 |

##### Table S4: Deaths of despair (thousands) by underlying cause of death group | England 2014-2022

| Year | Suicide | Alcohol use | Substance use | Mental health |
| --- | --- | --- | --- | --- |
| 2014 | 4.6 | 5.2 | 2.3 | 0.2 |
| 2015 | 4.7 | 5.4 | 2.7 | 0.2 |
| 2016 | 4.7 | 5.6 | 2.9 | 0.2 |
| 2017 | 4.9 | 5.9 | 3.0 | 0.2 |
| 2018 | 5.4 | 5.7 | 3.4 | 0.2 |
| 2019 | 5.4 | 6.0 | 3.6 | 0.2 |
| 2020 | 5.4 | 7.1 | 3.7 | 0.3 |
| 2021 | 5.4 | 7.7 | 3.9 | 0.2 |
| 2022 | 5.2 | 8.2 | 3.7 | 0.3 |
| % change 2014 - 2022 | +14.0% | +56.2% | +56.9% | +32.6% |

##### Table S5: Deaths from injury or poisoning of undetermined intent as a percentage of all deaths of despair

| Group | 2014 | 2022 | % change |
| --- | --- | --- | --- |
| all | 7.60% | 3.00% | 39.00% |
| women | 7.50% | 3.00% | 39.70% |
| men | 7.60% | 3.00% | 38.60% |
| most deprived | 5.80% | 2.60% | 44.60% |
| least deprived | 8.60% | 3.10% | 36.20% |

##### Table S6: Directly standardised (despair) mortality rates by year and quintile of deprivation with 95% confidence intervals | England 2014 – 2022

| year | quintile 1 | quintile 2 | quintile 3 | quintile 4 | quintile 5 |
| --- | --- | --- | --- | --- | --- |
| 2014 | 41.2 (39.9, 42.4) | 26.8 (25.8, 27.8) | 19.4 (18.6, 20.2) | 14.8 (14.1, 15.5) | 12.5 (11.8, 13.1) |
| 2015 | 41.9 (40.7, 43.2) | 27.7 (26.7, 28.7) | 21.0 (20.2, 21.9) | 16.6 (15.9, 17.3) | 12.8 (12.2, 13.5) |
| 2016 | 43.0 (41.7, 44.2) | 28.5 (27.5, 29.5) | 20.8 (19.9, 21.6) | 17.5 (16.7, 18.3) | 12.7 (12.1, 13.4) |
| 2017 | 44.7 (43.4, 46.0) | 29.5 (28.4, 30.5) | 21.6 (20.7, 22.5) | 16.9 (16.2, 17.7) | 13.7 (13.0, 14.4) |
| 2018 | 45.7 (44.4, 47.0) | 29.6 (28.6, 30.7) | 23.9 (23.0, 24.8) | 17.8 (17.0, 18.6) | 14.4 (13.7, 15.1) |
| 2019 | 47.5 (46.2, 48.8) | 31.2 (30.2, 32.3) | 23.6 (22.7, 24.5) | 18.6 (17.8, 19.4) | 14.8 (14.1, 15.5) |
| 2020 | 52.6 (51.2, 54.0) | 34.3 (33.2, 35.4) | 25.1 (24.2, 26.0) | 20.0 (19.2, 20.8) | 15.4 (14.7, 16.1) |
| 2021 | 53.1 (51.7, 54.5) | 35.8 (34.7, 36.9) | 26.9 (26.0, 27.9) | 20.7 (19.9, 21.5) | 15.9 (15.2, 16.6) |
| 2022 | 52.5 (51.1, 53.9) | 35.3 (34.2, 36.4) | 26.4 (25.4, 27.3) | 21.2 (20.3, 22.0) | 16.7 (16.0, 17.4) |
| per 100,000 population \| quintile 1 - most deprived | | | | | |

##### Table S7: Directly standardised (despair) mortality rates by year and condition group with 95% confidence intervals | England 2014 – 2022

| year | Alcohol use | Mental health | Substance use | Suicide |
| --- | --- | --- | --- | --- |
| 2014 | 9.6 (9.4, 9.9) | 0.3 (0.2, 0.3) | 4.3 (4.1, 4.5) | 8.3 (8.1, 8.6) |
| 2015 | 9.8 (9.5, 10.0) | 0.3 (0.3, 0.4) | 5.0 (4.8, 5.2) | 8.6 (8.3, 8.8) |
| 2016 | 10.1 (9.9, 10.4) | 0.3 (0.3, 0.4) | 5.2 (5.0, 5.4) | 8.4 (8.2, 8.7) |
| 2017 | 10.6 (10.3, 10.9) | 0.3 (0.2, 0.3) | 5.4 (5.2, 5.6) | 8.7 (8.5, 9.0) |
| 2018 | 10.1 (9.9, 10.4) | 0.3 (0.3, 0.4) | 6.1 (5.9, 6.3) | 9.6 (9.4, 9.9) |
| 2019 | 10.4 (10.2, 10.7) | 0.3 (0.2, 0.3) | 6.5 (6.3, 6.7) | 9.7 (9.4, 9.9) |
| 2020 | 12.5 (12.2, 12.8) | 0.4 (0.3, 0.4) | 6.8 (6.5, 7.0) | 9.5 (9.3, 9.8) |
| 2021 | 13.3 (13.0, 13.6) | 0.3 (0.3, 0.4) | 7.0 (6.8, 7.3) | 9.4 (9.2, 9.7) |
| 2022 | 14.1 (13.8, 14.4) | 0.4 (0.3, 0.4) | 6.6 (6.3, 6.8) | 9.0 (8.8, 9.3) |
| per 100,000 population | | | | |

##### Figure S1. Directly age-standardised mortality rates for deaths of despair per 100,000 population in England, overall, by sex and socioeconomic deprivation, 2014–2022. The upper panels reproduce the primary analysis presented in Figure 1 of the main manuscript. The lower panels repeat the analysis using an expanded definition of deaths of despair that includes deaths due to injuries and poisonings of undetermined intent. The similarity between the two analyses demonstrates that the principal findings are robust to this alternative case definition.

##### Figure S2. Directly age-standardised mortality rates for deaths of despair per 100,000 population among men and women, stratified by socioeconomic deprivation, England, 2014–2022.

Mortality rates are shown separately for men and women, comparing the most deprived and least deprived socioeconomic quintiles. The figure illustrates that the socioeconomic gradient is substantially greater among men than among women, while both groups experienced increases during the COVID-19 pandemic.

##### Figure S3: Sensitivity analysis excluding deaths among people aged 90 years or over and excluding deaths attributed to non-organic mental health conditions.

The primary analysis (left panel) is compared with two alternative analytical approaches: excluding deaths among people aged 90 years or over (centre panel) and excluding deaths attributed to non-organic mental health conditions (right panel). The similarity of the temporal trends across all three analyses demonstrates that the principal findings are robust to these alternative analytical assumptions.
